# Introducing Adjunct Medical Products and Artificial Intelligence Governance Gaps

**DOI:** 10.1016/j.mcpdig.2026.100373

**Published:** 2026-05-20

**Authors:** Mindy Nunez Duffourc, Stephen Gilbert, Courtney Nadeau

**Affiliations:** a Faculty of Law, Department of Private Law, Maastricht Law and Tech Lab, Maastricht University, Maastricht, The Netherlands; b Else Kröner Fresenius Center for Digital Health, Faculty of Business and Economics, TUD Dresden University of Technology, Dresden, Germany; c Group Research and Development, DNV, Høvik, Norway

The European Union (EU) has responded to the modern artificial intelligence (AI) boom with new rules that promise to balance the potential benefits and risks of AI-driven technologies.^1^ The 2023 General Product Safety Regulation (GPSR—regulation (EU) 2023/988) and the 2024 AI Act—regulation (EU) 2024/1689 include rules that aim to ensure the safety of certain categories of AI-driven products on the EU market. The 2024 revision of the product liability directive (PLD—directive (EU) 2024/2853) provides updated liability rules that can address harm caused by AI-driven products on the EU market. These general safety and liability rules operate together with sector-specific rules that govern AI-driven technologies in the health care sector; for example, the medical devices regulation (MDR—regulation (EU) 2017/745) and in vitro diagnostic medical devices regulation (IVDR—regulation (EU) 2017/746) govern market safety for AI-driven medical devices. Despite the EU’s recent efforts to govern AI safety, a rapidly expanding category of AI-driven technologies enters the market without sufficient oversight; we call them adjunct AI medical products (AMPs).

## Introducing AMPs

AMPs describe AI-based software products that (1) are not independently reviewed for safety before entering the market, (2) are integrated into the digital systems and infrastructure through which health care is organized and delivered, and (3) can pose considerable risks to patient safety. They can include systems that provide general medical information, facilitate patient scheduling, transcribe patient interactions, or send medication reminders (Table). Many AMPs fall outside of the legal scope of medical device regulations because their claimed intended purposes are informative or administrative, rather than clinical. Notably, according to the Medical Device Coordination Group (Guidance 2019-11), ‘the risk of harm to patients is not a criterion on whether the software qualifies as a medical device.’ If designed and used responsibly, AMPs can enhance health care efficiency, efficacy, and access.^2^^,^^3^ However, without sufficient market safety regulation, they can present serious patient safety risks. This can in turn expose developers and health care providers to liability^3^ and hinder the development and adoption of even safe and beneficial AMPs.

**Table:** Adjunct Medical Products: Types, Examples, and Regulatory Status

AMP Type	Purpose and Examples	General Regulatory Classification	Req. Safety Assessment
Operational process facilitators	Purpose: staff planning, emailing, and billing Examples: patient scheduling and messaging software used by medical professionals	Medical device (MDR)? No, no qualifying medical purpose General product (GPSR)? No, not intended for consumers High-risk AI (AI Act)? No, not in enumerated list	None
Medical education & knowledge systems	Purpose: providing evidence-based guidelines, literature summaries, and simulations not intended to benefit individual patients Examples: chatbots designed for medical professionals	Medical device (MDR)? No, no qualifying medical purpose General product (GPSR)? No, not intended for consumers High-risk AI (AI Act)? No, not in enumerated list	None
Minimal-risk medical devices	Purpose: supporting medication adherence for noncritical treatments Examples: software that sends personalized medication reminders and dosing instructions to patients	Medical device (MDR)? Yes, Class I medical device under the MDR General product (GPSR)? No, for safety risks regulated by MDR High-risk AI (AI Act)? No, does not require third-party conformity assessment under MDR	Self-assessment
High-risk in-house AI software	Purpose: diagnosis, treatment, monitoring, prognosis, or other medical intended purpose for the benefit of individual patients within a single institution Examples: software that calculates stroke risk based on patient journal data put into service under the in-house exemption	Medical device (MDR)? Yes General product (GPSR)? No, for safety risks regulated by MDR High-risk AI (AI Act)? No, does not require third-party conformity assessment under MDR	Self-assessment
Consumer health information products	Purpose: answering general health or health care-related questions or providing wellness information Examples: general or health-specific chatbots; fitness, menstrual, pregnancy trackers; mindfulness and breast self-examination apps	Medical device (MDR)? No, no qualifying medical purpose General product (GPSR)? Yes High-risk AI (AI Act)? No, not in enumerated list	Self-assessment
Health care access decision systems	Purpose: determining patient eligibility for health care services Examples: software that automates the dispatch of emergency services	Medical device (MDR)? No, no qualifying medical purpose General product (GPSR)? No, not intended for consumers High-risk AI (AI Act)? Yes	Self-assessment

Abbreviations: AI, artificial intelligence; AMP, adjunct medical products; GPSR, general product safety regulation; MDR, medical devices regulation; IVDR, in vitro diagnostic medical devices regulation; PLD, product liability directive; Req., required.

### Regulatory Uncertainty

Safety governance for AI technologies in the health sector depends largely on how they are legally classified (Table).^4^^,^^5^ AMPs often escape meaningful safety assessment under the current framework because there is no clear legal classification to trigger a pragmatic level of oversight that avoids both over-regulation and under-regulation. Currently, medical device regulation under the MDR or IVDR and AI technology regulation under the AI Act are the primary mechanisms for ensuring the safety of AI-driven medical technologies on the EU market;^4^ however, they are not well-suited for balancing risks and benefits of AMPs.

The MDR or IVDR governs AI technologies that are classified as medical devices, but this classification is only triggered when the developer intends for the technology to be used for the purpose of diagnosis, prevention, monitoring, prediction, prognosis, treatment or alleviation of medical conditions (MDR art 2(1)). As a result, many AI applications that may initially appear to have medical uses are not legally classified as medical devices. Medical devices do not include AMPs with wellness or purely administrative purposes (Table).^6^ The AMPs that provide medical information intended for health care professionals only, including generic diagnostic or treatment pathways that are not for the benefit of individual patients are also not medical devices (Table).^6^ This means AI-driven medical technologies with similar uses may be classified differently in the regulatory framework if they have different intended purposes. For example, a chatbot that answers questions about patient-specific risks using information in electronic health records is likely a medical device, whereas a chatbot that answers questions about risk to a specific cohort using data from medical literature is less likely to be a medical device.

The AI Act applies risk-based regulation focused primarily on high-risk AI systems across all sectors, including health care; however, high-risk AI classification applies only to specific categories of technologies. AI-driven medical devices that require independent regulatory review before market entry through the process of third party-conformity assessment are considered high-risk systems under the AI Act and thus require the most stringent pre-market review for compliance with both the MDR/IVDR and AI Act. On the contrary, those that fall in the lowest MDR/IVDR risk classes or that are developed in-house under the health institution exemption will generally not require third-party conformity assessment (MDR art 52[7]; IVDR art 48[10]). This results in additional categories of AMPs that avoid pre-market safety assessment by regulators under the current framework (AI Act art 6[1] and annex I) (Table).

The GPSR governs AI software that is classified as a general consumer product; it requires that all products on the market be “safe” (GPSR art 5). The GPSR does not require pre-market regulatory review but relies instead on manufacturers’ internal risk analysis to ensure product safety (GPSR art 9[2]). The GPSR’s risk assessment obligations apply unless certain risks are more specifically covered by other EU market safety laws (GPSR art 2). This requires developers to conduct a safety self-assessment with little to no regulatory guidance for consumer AMPs, like breast self-examination applications, that may not be medical devices or high-risk AI systems (Table).

The GPSR does not cover, however, AMPs intended only for professional use by health care providers, for example, professionally marketed chatbots that retrieve responses from medical literature and treatment guidelines (Table).^7^^,^^8^ This category of AMPs is most concerning as they enter the market without any safety assessment, either by developers or regulators because they are not governed as medical devices under the MDR or IVDR, as high-risk AI systems under the AI Act, or as consumer products under the GPSR (Figure).

**Figure:**
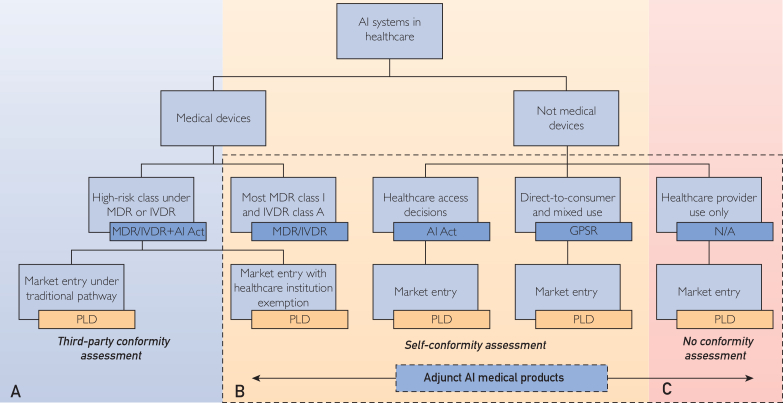
Overview of product safety and liability framework governing artificial intelligence (AI) systems for health AI systems in health care that are subject to regulatory review for market safety through third-party conformity assessment (A) that are AI medical products not subject to regulatory review through third-party conformity assessment but require self-conformity assessment (B) and that are AI medical products not subject to regulatory review and also do not require any conformity assessment (C). AI, artificial intelligence; GPSR, general product safety regulation; MDR, medical devices regulation; IVDR, in vitro diagnostic medical devices regulation; PLD, product liability directive.

### The Impact of Legislative Imbalance

The lack of clear legal rules governing AMP market safety creates uncertainty that also impacts the development and uptake of beneficial AMPs. This is because regardless of how AMPs are regulated for market safety, AMP developers and health care providers that use AMPs will face liability consequences when they cause harm. For health care providers, liability will be assessed under each Member States’ fault-based liability rules, and for manufacturers, the PLD provides harmonized strict product liability rules. Under the PLD, AMP developers will be liable for injuries caused by defective AMPs on the EU market. Products, including software (PLD art 4[1]), are defective if they do not “provide the safety that a person is entitled to expect or that is required under Union or national law” (PLD art 7). Although compliance with safety regulations, like those in the MDR/IVDR, AI Act, and GPSR, will not automatically shield developers from strict product liability, regulatory compliance can impact whether the AMP is considered defective (PLD art 7[f]). When market safety standards are lacking or ill-defined, it becomes difficult for manufacturers, health care providers, regulators, and courts to predict and assess the relevant levels of safety needed to prevent dangerous AMPs from entering the market or to determine (or avoid) liability for injuries caused by AMPs already on the market.

Legal uncertainty presents a 2-fold problem: (1) risks to patient safety from dangerous AMPs, and (2) barriers to the development of beneficial AMPs. First, AMPs that fall outside of the scope of clear safety regulation or rely solely on safety self-assessment or the voluntary application of risk management may not undergo sufficient evaluation to prevent risks of patient harm.^9^ Developers who are committed to providing safe and beneficial AMPs face barriers caused by legal uncertainty surrounding both safety and liability rules governing AMPs.^5^ Without a clear regulatory framework, developers must determine for themselves whether AMPs are sufficiently safe with little to no regulatory guidance. In addition, the lack of clear mechanisms to assess quality of competing AMPs can lead to market incentives that encourage a race to the bottom. The lack of regulatory clarity for AMPs means that it is up to health care providers and health systems to evaluate safety, increasing costs and presenting a substantial adoption barrier for health-technology companies aiming to access the European market.

### Measures to Promote Safe and Responsible AMPs

It is not practical to expect legal frameworks to provide complete clarity and coverage for all AI-driven health technologies, which span a wide range of potential uses, users, and design possibilities. Regardless of how they are regulated, defined, and classified, there will inevitably be a gray zone in which technologies evade specific regulatory coverage. However, AMPs have emerged as a rapidly growing group within this gray zone that demand more attention because they threaten the overall aims and spirit of the governing frameworks that promise safety and responsibility. It is challenging to respond to the lack of clarity for AMP safety regulation without fundamentally restructuring medical device regulation, for example by expanding intended purpose to encompass nonmedical technologies as a regulatory trigger for MDR/IVDR and AI Act coverage. We propose that such a fundamental restructuring of medical device regulations may not be required or even desirable for many AMPs, but that other, less disruptive, approaches might offer a promising path forward for safe and responsible AMP development. These include:

#### Expanding the Scope of the GPSR to Include AMPs Used Exclusively by Health Care Providers

These AMPs currently escape all pre-market safety regulation (Figure) and present a huge imbalance within the legal framework by removing market safety barriers and leading to overreliance on liability rules to compensate for injuries. Practically, this would require developers to conduct internal safety and risk assessments for AMPs that are considered Medical Education & Knowledge Systems (Table) to help improve both AMP safety and provider trust for this category of AMPs without subjecting them to overly burdensome pre-market review. It could trigger the demand for clear regulatory guidance and safety standards to facilitate and measure compliance for AMPs that require safety self-assessments under the GPSR.

#### Providing Regulatory Clarification on Legal Classifications for AMPs From the AI Office, the Medical Device Coordination Group, and Regulators Responsible for General Product Safety

Without a clear understanding of how AMPs are governed within the complex market safety framework, compliance becomes difficult and costly Currently, compliance and liability risks may lead especially cautious AMP developers to predict legal classifications that trigger high or impossible compliance burdens in areas of uncertainty and stall responsible innovation. Other AMP developers might misclassify AMPs in a way that avoids even minimal pre-market safety measures.

#### Clear Integration of Standards for Quality and Risk Management and Organizational Design for AMP Manufacturers or Deployers Into Safety and Defectiveness Assessments

For example, ISO 42001 includes a framework for managing organizational AI risks, and upcoming harmonized standards on AI quality and risk provide organizational measures that could prove suitable for AMPs.^10^ Regulators can provide guidance and promote risk-based approaches for AMP developers to limit the potential harms of their products.

#### Providing a Legal Basis for Including AMPs in Existing or new Regulatory Sandbox Frameworks

Regulatory sandboxes describe a transitional regulatory safe space for developers to conduct real-world AMP testing under increased supervision prior to full market access. They can help avoid disproportionate negative impacts of safety regulation on the pace of innovation. Frameworks for sandboxes are set out under the AI Act (and under the proposal for the revision to the MDR and IVDR) to allow pre-market exploration of regulatory challenges in a cooperative partnership between the regulatory bodies and the manufacturer, with input from wider stakeholders including health systems. This would be especially helpful for AMPs that present challenging questions regarding the future direction that regulatory guidance needs to take, or how regulatory bodies should apply standards for safety assessment, for example, when AMPs interact with other AI-enabled medical devices.^11^

These measures may help the EU strike the seemingly elusive balance between innovation and safety for AMPs. The governing framework for AMPs should also reflect a balance between harm prevention through safety regulation and compensation/deterrence through liability rules. Achieving this balance means avoiding overregulation of beneficial technologies while still fairly compensating individuals for injuries and deterring the development and marketing of unsafe AMPs.

## Potential Comepting Interests

The authors declare that they have no conflicts of interest to disclose.
